# A population-based survey on family intentions and fertility awareness in women and men in the United Kingdom and Denmark

**DOI:** 10.1080/03009734.2016.1194503

**Published:** 2016-06-24

**Authors:** Ditte Vassard, Camille Lallemant, Anders Nyboe Andersen, Nick Macklon, Lone Schmidt

**Affiliations:** a Department of Public Health, University of Copenhagen, DK-1014 Copenhagen K, Denmark;; b Complete Fertility Centre, Princess Anne Hospital, Coxford Road, Southampton, SO16 5YA, UK;; c The Fertility Clinic, Rigshospitalet, Copenhagen University Hospital, DK-2100 Copenhagen Ø, Denmark

**Keywords:** Comparative study, cross-sectional study, family intentions, fertility awareness, gender, population-based survey

## Abstract

**Background:**

Across several European countries family formation is increasingly postponed. The aims of the study were to investigate the desire for family building and fertility awareness in the UK and Denmark.

**Methods:**

A population-based internet survey was used among women (*n* = 1,000) and men (*n* = 237) from the UK (40%) and Denmark (60%). Data covered socio-demographics, family formation, and awareness of female age-related fertility. Data analysis used descriptive statistics and logistic regression analysis for studying associations between low fertility awareness and desired family formation.

**Results:**

The majority of all participants desired two or three children. Two-thirds of the childless participants desired a first child at 30+ years, and one-fifth of the women and one-third of the men desired a last child at age 40. Overall, 83% of women and 73% of men were aware that female fertility starts to decline around 25–30 years. Men had significantly lower fertility awareness. Women who underestimated the impact of age on female fertility were significantly more likely to have a desire or attempted their first child at a higher age.

**Conclusion:**

Even though the majority were aware of the age-related decrease in female fertility, most desired having children at an age when female fertility has declined. Women who were not sufficiently aware of the impact of advanced age were significantly more likely to have their first child at a higher age. There is a need for developing educational programs for women and men in order to increase the population’s knowledge of fertility and risk factors for infertility.

## Introduction

Across the developed world people are postponing parenthood, and in several Western European countries the mean age of first-time mothers is now around 28–30 years while fathers, on average, are around three years older (1). Studies have shown that the majority of childless women and men desire to become parents in the future (2–10), and most express a wish to have two or three children (2–4,6,7). However, a desire for parenthood does not equate to achieving parenthood, and among American childless 39-year-old women 73% desired a child in the future; but only 7% had one by age 45 (9). Habbema et al. (11) used a computer simulation model to show that couples who desire a two-child family with at least a 90% success rate should start at the latest at female age 27 years if couples did not intend to go through *in vitro* fertilization (IVF) treatment, and at 31 years if IVF could be accepted. For three children the age was 23 years and 28 years, respectively.

The causes for delayed childbearing are complex (12). However, the relationship between rise in educational participation resulting in later ages at end of education and later parenthood has, based on data from the United Kingdom and France, been shown to be almost certainly causal (13,14). Furthermore, a number of studies have shown that a substantial proportion of both women and men underestimate the impact of female age-related decline in fertility. These results have been reported from population-based studies (15), childless individuals (5,16), university student samples (2–5,7,8,10,17,18), among those trying to conceive for >6 months (19), women who conceived after IVF treatment (20), and women working in the health care sector (21). Hence, delayed childbearing is not necessarily an informed decision-making but could be a less conscious choice associated with lack of knowledge regarding the impact of female age on fertility (22).

United Kingdom and Denmark have differences as well as similarities in fertility patterns, and, furthermore, public fertility campaigns regarding fertility awareness prior to this study have been conducted in the UK but not in Denmark. Hence, we found it of interest to conduct a comparative study on family formation intentions and the knowledge of decline in fertility associated with increase in female age. In both countries, women and men have in recent decades increasingly postponed parenthood. A recent register-based study from Denmark showed, however, that the postponement of births for men seems about to end (year 2010) whereas the postponement of childbirth for women is still ongoing (23). At the time of data collection for this study (2012–2013) the mean age of first-time mothers was 27.9 years in the UK in 2011 (24) and 29.1 in Denmark (25). However, in the UK 24% of the mothers were <25 years old (24) compared to Denmark where only 12% of children were born of mothers <25 years (25). Regarding fertility awareness, public campaigns have been conducted in the UK covering information on increasing female age, smoking, and obesity as risk factors for infertility, while in Denmark such a campaign for the first time was recently established in October 2015 in the municipality of Copenhagen after this study was performed.

The aims of the present study were to investigate the desire for family building as well as awareness of female age-related decline in fertility among women and men in the United Kingdom and Denmark. We additionally investigated whether there were gender differences or differences between participants from the two countries.

## Material and methods

### Study population and procedures

The Ethics and Research Governance Committee of the University of Southampton, UK approved the present study. In Denmark, ethical approval for survey studies not including biomedical data are not required due to the legislation. The internet-based survey was advertised electronically in the UK and Denmark from September 2012 to September 2013 through general websites and sites from different educational institutions aimed at achieving representation from all adult age groups in the population, women and men, and with different educational background (www.mycompletefamily.co and www.mycompletefamily.dk). Furthermore, the survey was advertised physically in leaflets at different educational institutions, sports clubs, and libraries in Southampton, UK and in the capital region in Denmark. There were no incentives for participating. In all, 1,237 participants responded to the survey (1,000 women and 237 men). Forty percent (*n* = 555) were from the United Kingdom and 60% (*n* = 682) from Denmark.

### Measures

*Family formation.* Participants who were parents were asked about their present number of children, whether they desired more children in the future, and at what age they attempted to become a parent. Participants were asked about their own attempted age for parenthood, not their partner’s age. Present number of children and a further desired number of children were combined to an overall total number of desired children.

*Family intentions.* Participants who were not parents were asked whether they want to have children, and, if yes, how many children; and their own desired age at first child and last child.

*Awareness regarding female age and fertility.* Questions included were: (1) ‘At what age, on average, do you think a woman’s fertility begins to decline?’ (20/25/30/35/40/45 years); (2) ‘From what age do you think this decline may make it difficult for a woman to have a baby?’ (25/30/35/40/45 years); (3) ‘What are the chances that a woman of 30 years of age will become pregnant in one year of having unprotected sex?’ (10%–19%/20%–39%/40%–59%/60%–79%/80%–100%); (4) ‘And what if she is 40 years old?’ (10%–19%/20%–39%/40%–59%/60%–79%/80%–100%).

*Socio-demographics.* We recorded age, sex, marital status (married/cohabitating/not cohabitating/single/widowed), whether the participant had full-time/part-time paid work/no work, and educational level. As the educational system differs between UK and Denmark response categories for UK participants were: left school without GCSEs/left school with GCSEs/currently doing A-levels/completed A-levels/currently doing undergraduate studies/completed university degree at undergraduate level/currently doing a postgraduate degree/completed postgraduate degree/others (specify). For Danish participants: completed school education (9th grade/10th grade/GCSE level A) or currently doing school education (GCSE level A)/currently doing or completed further training (no vocational training/semi-skilled/skilled/short further/mid further/long further/others (specify)).

### Data analyses

SAS was used for all statistical analyses. Descriptive analyses were conducted using the chi-square test for studying differences between men and women and between countries. The significance level was determined as *P*-value < 0.05. Data were analyzed using logistic regression analyses unadjusted and adjusted for relevant variables in order to (1) identify factors associated with low fertility awareness and intended time to start a family, and whether having low fertility awareness was associated with desired age or age of attempt for a first child, and (2) investigate whether total number of desired children differed between childless and not childless participants. Participants with missing responses on key variables were excluded from the population in preparation for analyses.

In order to compare across countries regarding educational level, we integrated the response categories into: (1) GCSE/A-levels (UK) and finished school education/currently GCSE A-level (Denmark), (2) undergraduate (UK) and short- or medium-length further education (Denmark), (3) postgraduate (UK) and long further education (Denmark), and (4) vocational training (UK) and semi-skilled training (Denmark).

## Results

Table 1 presents socio-demographic characteristics of our sample (*n* = 1,237), and Table 2 presents family building intentions (childless participants) and family establishment and desires (participants who are parents). Respondents from Denmark had undergone a longer education (56% postgraduate versus 33%), were more often students (41% versus 17%), and more often single (24% versus 13%) when compared with respondents from the United Kingdom. Respondents from Denmark were less often employed full time (37% versus 56%), and childless participants less often desired their first child at age 35 or later (15% versus 28%) than respondents from the United Kingdom. The female respondents had higher education (47% postgraduate versus 37%), were less often employed full time (42% versus 58%), and the childless female participants less often desired their first child at age 35 or later (17% versus 31%) than the male respondents. Overall 85% of the childless participants desired to have children in the future. The majority of both childless participants and parents desired to have two or three children (84% among childless versus 75% among parents), and only few desired one child or four children or more (8% and 7%, respectively). On average, childless participants desired fewer children in total compared to participants being parents (mean total number of children 2.24 (SD 0.81) and 2.80 (SD 1.02), respectively, *P* < 0.001). Logistic regression analysis adjusted for age, gender, and country did not change this result. One-fifth of the women and one-third of the men desired a last child at age 40. Among the childless participants more men than women desired a last child by age 40 (31% versus 21%).

**Table 1.** Socio-demographic characteristics of the study population (*n* = 1,237).

		All *n* (%)	Women (*n* = 1,000) *n* (%)	Men (*n* = 237) *n* (%)	Chi-square *P* value	DK (*n* = 682) *n* (%)	UK (*n* = 555) *n* (%)	Chi-square *P* value
Age	18–24	253 (22)	207 (22)	46 (21)	0.10	188 (28)	65 (13)	<0.0001
	25–34	504 (43)	422 (44)	82 (38)		322 (47)	182 (37)	
	35–44	292 (25)	236 (25)	56 (26)		125 (18)	167 (34)	
	45+	128 (11)	95 (10)	33 (15)		47 (7)	81 (16)	
	*Missing*	*60*						
Education	GCSE/A-levels	164 (14)	110 (11)	54 (23)	<0.0001	30 (5)	134 (25)	<0.0001
	Undergraduate	417 (35)	332 (34)	85 (36)		203 (31)	214 (40)	
	Postgraduate	544 (45)	457 (47)	87 (37)		365 (56)	179 (33)	
	Vocational training	71 (6)	64 (7)	7 (3)		58 (9)	13 (2)	
	*Missing*	*41*						
Working status	Employed full time	541 (45)	406 (42)	135 (58)	<0.0001	242 (37)	299 (56)	<0.0001
	Employed part time	159 (13)	148 (15)	11 (5)		67 (10)	92 (17)	
	Student	356 (30)	297 (31)	59 (25)		267 (41)	89 (17)	
	Not employed	134 (11)	106 (11)	28 (12)		76 (12)	58 (11)	
	*Missing*	*47*						
Marital status	Married	540 (45)	415 (43)	125 (54)	0.00	200 (30)	340 (63)	<0.0001
	Cohabitating	320 (27)	285 (29)	35 (15)		225 (34)	95 (18)	
	Not cohabitating	108 (9)	85 (9)	23 (10)		75 (11)	33 (6)	
	Single	227 (19)	180 (19)	47 (20)		159 (24)	68 (13)	
	Widowed	3 (0)	3 (0)	0 (0)		1 (0)	2 (0)	
	*Missing*	*39*						
Have children	Yes	585 (48)	477 (49)	108 (46)	0.49	286 (42)	299 (56)	<0.0001
	No	629 (52)	503 (51)	126 (54)		392 (58)	237 (44)	
	*Missing*	*23*						
Want (more) children	Yes	664 (68)	587 (68)	77 (67)	0.75	420 (68)	244 (67)	0.28
in the future	No	223 (23)	198 (23)	25 (22)		132 (22)	91 (25)	
	Don’t know	92 (9)	79 (9)	13 (11)		63 (10)	29 (8)	
	*Missing*	*258*						

**Table 2.** Family intentions and family formation among childless participants (*n* = 629) and participants having children (*n* = 585).

		All *n* (%)	Women (*n* = 1,000) *n* (%)	Men (*n* = 237) *n* (%)	Chi-square *P* value	DK (*n* = 682) *n* (%)	UK (*n* = 555) *n* (%)	Chi-square *P* value
Questions for those without children (*n* = 629)								
Want to have children	Yes	471 (85)	411 (86)	60 (76)	0.00	344 (89)	127 (75)	<0.0001
	No	33 (6)	22 (5)	11 (14)		12 (3)	21 (12)	
	Don't know	52 (9)	45 (9)	7 (9)		31 (8)	21 (12)	
	*Missing*	*72*						
Desired number of children^a^	1	39 (8)	30 (7)	9 (15)	0.10	21 (6)	18 (15)	0.03
	2	261 (56)	226 (55)	35 (59)		193 (56)	68 (55)	
	3	131 (28)	120 (29)	11 (19)		101 (29)	30 (24)	
	4+	36 (8)	32 (8)	4 (7)		28 (8)	8 (6)	
	*Missing*	*4*						
Desired age first child	25	155 (28)	133 (30)	22 (23)	0.02	104 (30)	51 (26)	0.00
	30	282 (52)	239 (53)	43 (45)		190 (55)	92 (46)	
	35	95 (17)	68 (15)	27 (28)		49 (14)	46 (23)	
	40	13 (2)	10 (2)	3 (3)		4 (1)	9 (5)	
	*Missing*	*108*						
Desired age last child	25	10 (2)	6 (1)	4 (4)	0.02	2 (1)	8 (4)	0.00
	30	73 (13)	58 (13)	15 (16)		48 (14)	25 (13)	
	35	335 (62)	289 (64)	46 (49)		228 (66)	107 (55)	
	40	125 (23)	96 (21)	29 (31)		69 (20)	56 (29)	
	*Missing*	*110*						
Questions for those with children (*n* = 585)								
Number of children	1	216 (37)	179 (38)	37 (35)	0.57	120 (42)	96 (33)	0.00
	2	241 (42)	192 (41)	49 (46)		99 (35)	142 (48)	
	3+	121 (21)	101 (21)	20 (19)		66 (23)	55 (19)	
	*Missing*	*7*						
Want more children	Yes	236 (50)	214 (50)	22 (52)	0.35	117 (43)	119 (60)	0.00
	No	190 (41)	176 (41)	14 (33)		120 (44)	70 (35)	
	Don’t know	42 (9)	36 (8)	6 (14)		33 (12)	9 (5)	
	*Missing*	*117*						
Additional number of children	1	79 (62)	70 (64)	9 (53)	0.03	73 (63)	6 (55)	0.48
wanted^b^	2	29 (23)	27 (25)	2 (12)		27 (23)	2 (18)	
	3+	19 (15)	13 (11)	6 (35)		16 (14)	3 (27)	
	*Missing*	*109*						
Total number of children wanted^c^	1	26 (8)	22 (8)	4 (13)	0.02	15 (6)	11 (14)	0.01
	2	141 (45)	130 (46)	11 (35)		98 (42)	43 (54)	
	3	262 (30)	112 (39)	9 (29)		99 (42)	22 (28)	
	4+	65 (7)	21 (7)	7 (23)		24 (10)	4 (5)	
	*Missing*	*269*						
Age attempting to have first child	24	145 (27)	126 (30)	19 (18)	0.00	64 (25)	81 (30)	<0.0001
	25–29	222 (42)	183 (43)	39 (38)		134 (52)	88 (32)	
	30–34	117 (22)	88 (21)	29 (28)		47 (18)	70 (26)	
	35+	46 (9)	30 (7)	16 (16)		12 (5)	34 (12)	
	*Missing*	*124*						

a Among *n* = 471 that want children.

b Among *n* = 236 that want more children.

c Number of children plus number of planned/wanted children in the future.

As shown in Table 3 there were gender differences related to fertility awareness. More men (18%) than women (8%) stated that the decline in female fertility started to decline at 35 years of age or after. Also more men (35%) than women (18%) stated that it becomes difficult for a woman to have a child at age 40 or later. Women and men had quite similar estimations of chances of pregnancy within one year in 30-year-old women, but men (36%) more often overestimated the chances of conception within one year at 40 years of age than did women (21%). On the other hand, 22% of women and 44% of men reported that it would be difficult for a woman to have a child at age 25 or 30, thus earlier than the correct age of 35. In general, more participants from the UK had a poorer knowledge on female age-related decline in fertility when compared to Danish participants.

**Table 3.** Fertility awareness among women and men.

Categories	All *n* (%)	Women (*n =* 974–978) %	Men (*n =* 235) %	Chi-square *P* value	DK (*n =* 682) %	UK (*n =* 555) %	Chi-square *P* value
At what age, on average, do you think a woman’s fertility begins to decline?							
20	120 (10)	10	9	<0.0001	0	22	<0.0001
25^a^	546 (45)	47	38		53	36	
30^a^	431 (36)	36	35		36	35	
35	66 (5)	6	5		10	0	
40	23 (2)	0	10		0	4	
45	27 (2)	2	3		2	3	
*Missing*	*24*						
From what age do you think this decline will make it difficult for a woman to have a child?							
25	48 (4)	3	6	<0.0001	3	5	<0.0001
30	272 (22)	19	38		20	25	
35^a^	633 (51)	60	20		64	38	
40	153 (12)	10	23		0	28	
45	107 (9)	8	12		13	4	
*Missing*	*24*						
What are the chances that a woman of 30 years of age will become pregnant after one year of unprotected sex?							
10%–19%	22 (2)	2	2	0.00	1	2	0.04
20%–39%	253 (20)	22	16		23	18	
40%–59%	446 (36)	38	32		38	35	
60%–79%^a^	379 (31)	31	34		30	33	
80%–100%	112 (9)	8	15		7	11	
*Missing*	*25*						
And what if she is 40 years of age?							
10%–19%	545 (45)	48	32	<0.0001	52	36	<0.0001
20%–39%^a^	376 (31)	31	33		27	36	
40%–59%	177 (15)	13	20		11	19	
60%–79%	98 (8)	7	13		8	8	
80%–100%	13 (1)	1	3		1	1	
*Missing*	*28*						

a Correct response according to reproductive epidemiological studies.

Table 4 shows unadjusted and adjusted logistic regression analyses indicating that respondents who overestimated the chance of pregnancy in a 30-year-old woman were more likely to have attempted or to desire their first child at a higher age (odds ratio (OR) 2.8, 95% confidence interval (CI) 1.4–5.4 for the category 25–29 years; OR 3.3, 95% CI 1.5–7.1 for the category 30–34 years) compared to those who did not overestimate the chance. Also, an insignificant stepwise increase in age of attempt or desire for a child was observed in those respondents who estimated the age of difficulty to become pregnant later than 35 years of age. Separate analyses for men indicated stronger associations between fertility awareness and timing of intended family formation, although results were insignificant due to a smaller number of men (data not shown).

**Table 4.** Unadjusted and adjusted odds ratio (OR) and 95% confidence intervals (CI) for the associations between lower fertility awareness and age of desired/attempted first child (*n* = 1,035).

	Categories	*n* (%)	Low fertility awareness *n* (%)	Unadjusted OR (95% CI)	Adjusted for age	Adjusted for age, sex, and education
Estimates the female fertility decline later than 30 years of age						
Age desire/attempt	≤24	275 (28)	38 (40)	1.0	1.0	1.0
	25–29	468 (47)	40 (43)	0.6 (0.4–0.9)^a^	0.9 (0.5–1.5)	1.1 (0.6–1.8)
	30–34	195 (20)	12 (13)	0.4 (0.2–0.8)^a^	0.8 (0.4–1.7)	1.0 (0.5–2.1)
	35+	54 (5)	4 (4)	0.5 (0.2–1.5)	0.7 (0.2–2.3)	0.9 (0.3–2.9)
Estimates female age when it becomes difficult to have a child as later than 35 years of age						
Age desire/attempt	≤24	275 (28)	58 (28)	1.0	1.0	1.0
	25–29	468 (47)	89 (43)	0.9 (0.6–1.2)	1.1 (0.8–1.7)	1.3 (0.9–1.9)
	30–34	195 (20)	43 (21)	1.1 (0.7–1.7)	1.5 (1.0–2.5)	1.6 (1.0–2.6)
	35+	54 (5)	15 (7)	1.5 (0.8–2.8)	1.8 (0.9–3.6)	1.9 (0.9–3.9)
Overestimates probability of pregnancy in a woman of 30 years of age						
Age desire/attempt	≤24	275 (28)	12 (13)	1.0	1.0	1.0
	25–29	468 (47)	46 (51)	2.7 (1.4–5.2)^a^	2.9 (1.5–5.6)^a^	2.8 (1.4–5.4)^a^
	30–34	195 (20)	26 (29)	3.6 (1.8–7.3)^a^	3.5 (1.7–7.5)^a^	3.3 (1.5–7.1)^a^
	35+	54 (5)	7 (8)	3.2 (1.2–8.5)^a^	2.8 (1.0–8.0)	2.5 (0.9–7.3)^a^
Overestimates probability of pregnancy in a woman of 40 years of age						
Age desire/attempt	≤24	275 (28)	64 (28)	1.0	1.0	1.0
	25–29	468 (47)	105 (46)	1.0 (0.7–1.4)	1.2 (0.8–1.7)	1.2 (0.8–1.8)
	30–34	195 (20)	46 (20)	1.0 (0.7–1.5)	1.2 (0.8–1.9)	1.2 (0.7–1.9)
	35+	40 (5)	14 (6)	1.1 (0.6–2.2)	1.1 (0.5–2.2)	1.0 (0.5–2.0)

Also tested in the models: Partner status (married/cohabitant/single and other), have children (yes/no).

a *P* value <0.05.

## Discussion

### Family building

Overall, the majority of participants desired establishing a family of two or three children—either they already were parents or they desired to become parents in the future. This is in line with previous studies from a number of Western countries, California (8), Canada (16), Denmark (21), Finland (7), Italy (10), and Sweden (2–4). It is remarkable in this study that nearly one-fifth of the women and nearly one-third of the men desired a first child at age 35 or older, and similar proportions of women and men desired a last child at age 40 years—ages when female fertility has declined or greatly declined. As men on average have children with a partner two to three years younger than themselves (26), desiring a last child at male age 40 will on average be associated with a partner being around 37–38 years old. The proportion desiring a last child at 40 years of age is comparable to results from other studies (2,3). As mentioned in the introduction section, Habbema et al. (11) have made computer simulations on fertility showing that couples should start family formation at the latest at female age 31 years and at 28 years for a 90% chance of having a two-child or three-child family, respectively, if IVF treatment is included as an option. Leridon (27) has shown, based on computer simulation models, that assisted reproduction technology (ART) treatment only makes up for half of the births lost by postponing a first attempt of pregnancy from female age 30 to 35, and less than 30% if postponed from 35 to 40 years. However, studies show that many men and women believe that assisted reproduction technology treatment can restore fertility (16,20,28,29).

Significantly, fewer childless men compared to childless women desired to have children in the future. In Denmark, the proportion of children born without a man registered as father has increased from around 1%–1.5% during 1980–2005 to around 3% during 2005–2010 (23), and a part of this increase is due to access in both the public and private health care sector for single women to use sperm donation since 2007. Among single women using semen donation two-thirds reported having had a partner with whom they desired to build a family. However, 38% of the partners already were fathers and one-third did not want more children (30).

### Fertility awareness

Overall, 83% of the female and 73% of the male study participants from the UK and Denmark correctly reported that female fertility starts to decline around 25–30 years. The majority of women were aware that it becomes difficult for women from age 35 to have a child, but more than one-third of men overestimated the age when it becomes difficult for women to have a child. In general, study participants from the UK and Denmark reported similar knowledge regarding the impact of female age on fertility. However, significantly more participants from the UK expected child-bearing to become difficult at an older age than the correct age of 35 years. It is interesting that fertility awareness regarding the female age-related decline in fertility is similar in the UK and Denmark, as public fertility campaigns before this study was performed had been conducted only in the UK. However, in both countries advanced age and fertility have in recent years been a frequent topic in the mass media. Further, we found that a substantial proportion underestimated the chances of becoming pregnant after one year. This could indicate that the public awareness of age-related decline in fertility also leads to overemphasizing the problem and correct knowledge is still needed. Compared to a previous Australian study among the general population where only 26% correctly stated that female fertility starts to decline before the age of 35 (15), it seems that the knowledge on the age-related decline in female fertility is higher in our study.

In accordance with previous studies (2,5,7,13,15) we found that men had significantly lower fertility awareness than women on almost all parameters. Further, among men fertility awareness was not significantly associated with age of desiring or attempting to have the first child. The latter finding, although based on a limited number of survey respondents, could indicate that factors other than fertility awareness and knowledge might have a larger influence on the decision about when to have children. This could be career concerns in connection with maternity or paternity leave and practical challenges of having small children while wishing to prioritize work, the issue of finding the right partner, that people want to finish studying before having children, or the desire for financial stability before having children, which has been shown in previous studies as important factors for family building (2,5,21,31).

### Strengths and limitations

An internet-based survey is an easy way of reaching a target study group of younger participants as the great majority of the younger population will be online. Furthermore, responding to an online survey is easy and simple compared to responding to a mailed questionnaire or an interview survey. On the other hand, the limitations include that the study population is not statistically representative of the background population, identities of respondents cannot be confirmed, and with an internet-based data collection it is not possible to calculate a response rate or conduct any non-responder analysis. In this study, 43% of the respondents were in the age group 25–34 years, which is the age when most people desiring children start to establish their family. Hence, fertility awareness in this age group is especially relevant. It is widely recognized that surveys with self-selection of respondents most often attract respondents with a higher education. Furthermore, it is possible that this study attracted participants having a particular interest and knowledge within fertility awareness and risk factors for reduced fertility. If this is the case, the results presented are probably representing a best-case scenario, and a representative population-based study would possibly have shown a higher proportion of participants having insufficient fertility awareness knowledge. As described in the methods section, attempts were made to attract respondents from all educational groups. Due to the selected group of respondents, it would be expected that including more people from groups with a shorter further training or shorter education and including a larger proportion of men would reveal a higher prevalence of low fertility awareness than what is found in this study.

### Clinical and societal implications

An important question when discussing delayed childbearing and age-related infertility is: How far can education of the public help us? Studies investigating effects of information about fertility awareness and about reproductive life plan (RLP)-based information are few. RLP is a health-promoting tool recommended by the US Centers for Disease Control and Prevention (CDC). The recommendation is to screen women and men for their intentions to have/not to have children in the short and long term in order to improve preconceptional health and decrease unintended pregnancies and adverse pregnancy outcomes (32). In Sweden, midwives are responsible for approximately 90% of all contraceptive counseling, and Stern et al. (32) investigated Swedish midwives’ experiences with RLP. The midwives generally adopted RLP in the counseling, had positive experiences using this tool, and reported that most women were interested and happy to be asked about their reproductive plans. Furthermore, Stern et al. (33) conducted a randomized controlled trial among young women visiting a health care center and showed that significantly more women in the intervention group were inclined to desire a last child earlier in life compared to non-intervention groups. In a Canadian study, Daniluk and Koert (34) showed a significant increase in knowledge of fertility and ART immediately after an online educational intervention, but six months later the participants’ knowledge, especially for the men, returned to baseline levels. It is of importance to identify health events where young people can be motivated to reflect on their family formation intentions. ‘Teachable moments’ are ‘naturally occurring health events thought to motivate individuals to spontaneously adopt risk-reducing health behaviors’ (35, p. 156). It is reasonable to believe that young people seeking contraceptive counseling is a naturally occurring health event suitable for reflections on RLP and hence providing an opportunity for young people to gain fertility awareness and include knowledge on infertility risk factors in their decision regarding timing of family formation. Besides increasing the population’s fertility awareness, it is of importance that politicians and other stakeholders continuously work for developing family-friendly societies. In industrialized countries a substantial proportion of women and men are highly educated, and most often both partners want to stay in the labor market also when becoming parents (36). Hence, it is highly important for a society to support universal possibilities for parental leave, for combining work and parenthood, as well as combining education and parenthood, among other things by having sufficient access to good-quality child day care. Mills et al. (12) have in a review study shown that policies aiming at ‘reducing the incompatibility between work and mother roles (e.g. maternity leaves, childcare, early education) are more effective and lead to younger ages at first birth’ (p. 857) compared to more mixed empirical results regarding policies involving cash and indirect benefits for parents.

## Conclusion

In conclusion, we found relatively adequate awareness of female age-related decline in fertility among the participants from the UK and from Denmark. However, men had significantly lower fertility awareness compared to women. Results from this study are in general similar to previous fertility awareness studies based on a number of different study populations and conducted in different countries. As results are similar across different study populations we believe there is a necessity to develop and evaluate educational programs for women and men in order to increase the population’s knowledge of fertility, risk factors for infertility, and knowledge regarding success rates of medically assisted reproduction treatment. Knowledge about fertility will enable people to make an informed and conscious choice about how long they will risk postponing childbearing and potentially reduce the risk of infertility associated with increasing age. Also the conditions offered by society and social circumstances are important to explore further. The aim is that among those people who desire a family, as many people as possible are able to achieve their desired family size.
